# Development of Small-Molecule MERS-CoV Inhibitors

**DOI:** 10.3390/v10120721

**Published:** 2018-12-17

**Authors:** Ruiying Liang, Lili Wang, Naru Zhang, Xiaoqian Deng, Meng Su, Yudan Su, Lanfang Hu, Chen He, Tianlei Ying, Shibo Jiang, Fei Yu

**Affiliations:** 1 College of Life and Science, Hebei Agricultural University, Baoding 071001, China; ruiyingliang@outlook.com (R.L.); dengxiaoqian0926@hotmail.com (X.D.); sumeng123@hotmail.com (M.S.); suyudan123@hotmail.com (Y.S.); hlf0519@hotmail.com (L.H.); hechen285@hotmail.com (C.H.); 2 Research Center of Chinese Jujube, Hebei Agricultural University, Baoding 071001, China; yywll@hebau.edu.cn; 3 Department of Clinical Medicine, Faculty of Medicine, Zhejiang University City College, Hangzhou 310015, China; zhangnr@zucc.edu.cn; 4 Key Laboratory of Medical Molecular Virology of MOE/MOH, School of Basic Medical Sciences, Fudan University, Shanghai 200032, China

**Keywords:** MERS-CoV, mechanism of action, small-molecule inhibitor

## Abstract

Middle East respiratory syndrome coronavirus (MERS-CoV) with potential to cause global pandemics remains a threat to the public health, security, and economy. In this review, we focus on advances in the research and development of small-molecule MERS-CoV inhibitors targeting different stages of the MERS-CoV life cycle, aiming to prevent or treat MERS-CoV infection.

## 1. Introduction

**Figure 1:**
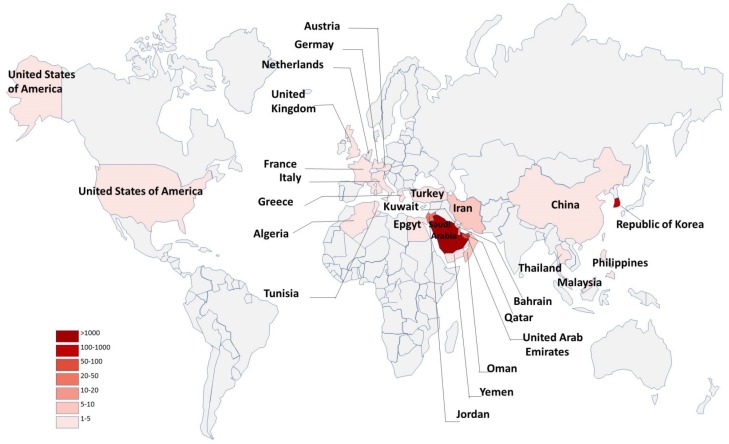
Summary of morbidity statistics with country- and quarter-level panel data.

Middle East respiratory syndrome coronavirus (MERS-CoV) has posed a serious threat to public health worldwide because it can cause severe respiratory disease in humans with high mortality (about 36%) [1]. As of 27 November 2018, a total of 2266 human MERS-CoV infections with 804 deaths had been reported from 27 countries in the Middle East, North Africa, Europe, Asia, and North America to the World Health Organization (WHO), with 83% reported by the Kingdom of Saudi Arabia (Figure 1) (https://www.who.int/emergencies/mers-cov/en/).

Phylogenetic and sequencing data strongly suggest that MERS-CoV belongs to the C-lineage of the genus betacoronavirus, the first known lineage C betacoronavirus associated with human infections [2]. The clinical features of MERS-CoV infection range from asymptomatic infection to rapidly progressive acute hypoxemic respiratory failure and extrapulmonary organ dysfunction [3,4,5]. At present, no effective vaccine or therapeutics are available for the prevention or treatment of MERS-CoV infection [6,7,8]. However, many basic and clinical studies on anti-MERS-CoV agents have been completed or are ongoing. In this review, we focus on current progress in the research and development of small-molecule MERS-CoV inhibitors, either peptides or compounds, targeting different stages of the MERS-CoV life cycle, aiming to prevent or treat MERS-CoV infection.

## 2. MERS-CoV Life Cycle and Potential Targets for the Development of Small-Molecule Inhibitors Against MERS-CoV Infection

MERS-CoV enters host cells through two pathways. The first involves plasma membrane fusion, which relies on spike (S) protein activation by secreted or surface proteases, such as the transmembrane protease serine 2 (TMPRSS2) and the human airway trypsin-like protease (HAT). The second involves endosomal membrane fusion, in which spike protein activation is facilitated by the pH-dependent endosomal protease cathepsin L (CTSL) [9,10]. The spike protein plays a key role in MERS-CoV attachment to host cells and virus-cell membrane fusion [11]. It contains 1353 amino acids within the viral envelope in trimeric state [12]. Spike protein consists of S1 and S2 subunits. The S1 subunit contains the receptor binding domain (RBD), while the S2 subunit contains the fusion peptide (FP), a long heptad repeat 1 domain (HR1) and a short heptad repeat 2 domain (HR2) [13,14]. MERS-CoV enters the host cell by binding the viral particle via the RBD in spike protein to the cellular receptor dipeptidyl peptidase-4 (DPP4) on the surface of the host cell [12,15]. Then, S2 changes its conformation and inserts its FP into the plasma membrane, or the endosomal membrane if the virion is in the endosome. The HR2 binds to the HR1 to form a six-helix bundle (6-HB) fusion core, which brings viral and cell membranes into close apposition for fusion [14,16,17]. During this process, RBD, DPP4, HR1, HR2, and the related proteases, e.g., HAT and TMPRSS2, can all serve as targets for the development of MERS-CoV fusion/entry inhibitors.

**Figure 2:**
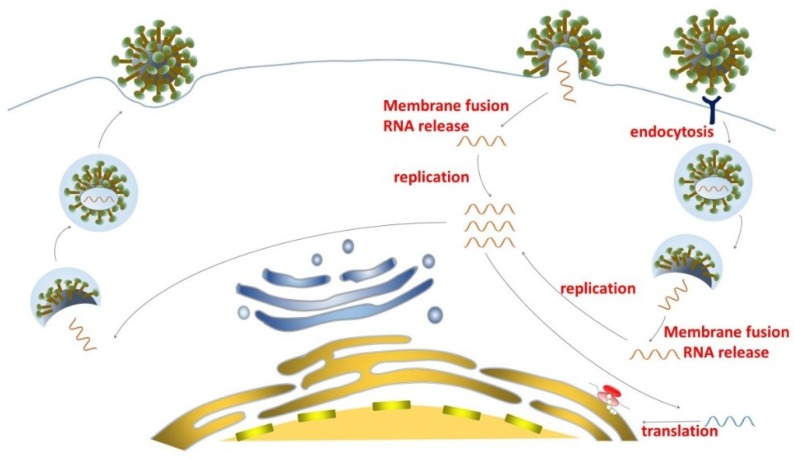
Schematic diagram of Middle East respiratory syndrome coronavirus (MERS-CoV) infection. MERS-CoV enters host cells by plasma membrane fusion (membrane fusion) or endosomal membrane fusion (endocytosis), and then releases the viral RNA into the cytoplasm. The RNA genome is replicated and viral proteins are produced. The progeny virus is generated and released from the infected cells.

After MERS-CoV entry into the host’s cells, the positive RNA genome is translated in the cytoplasm. The genome can be translated into two polyproteins: ppla and pplb, which are cleaved into 16 nonstructural proteins by PL_pro_ (papain-like protease) and 3CL_pro_ (3-chymotrypsin-like protease). Hence, the proteases that are critically important for MERS-CoV replication can also be considered as targets for developing MERS-CoV replication inhibitors. However, information about the enzymes required for producing more genome copies and subgenomic mRNA for virus replication is limited. Then, the RNA genome and structural proteins are packaged into viral particles in host cells, and the progeny virus particles are finally released from host cells (Figure 2). Although these steps can also be used as targets for the development of MERS-CoV maturation-and-release inhibitors, no such inhibitors have been reported so far.

## 3. Current Small-Molecule Inhibitors Against MERS-CoV Infection and Their Mechanisms of Action

### 3.1. MERS-CoV Entry Inhibitors

MERS-CoV S protein plays a key role in mediating virus entry into host target cells. This process includes binding to host receptors, viral fusion, and final entry into host cells. MERS-CoV pseudovirus expressing S protein, which allows for single-cycle infection in cells expressing receptor DPP4, can be used for screening MERS-CoV fusion/entry inhibitors.

**Figure 3:**
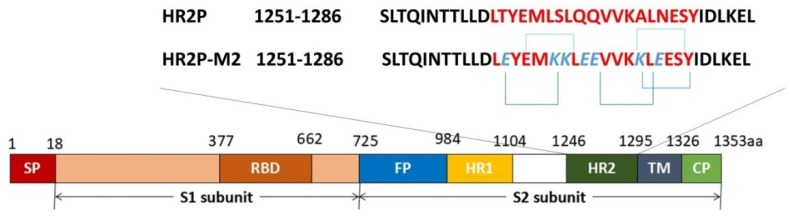
Schematic representation of MERS-CoV S (spike) protein S1 subunit and S2 subunit. RBD, receptor binding domain; FP, fusion peptide; HR1, heptad repeat 1 domain; HR2, heptad repeat 2 domain; TM, transmembrane domain; CP, cytoplasmic domain. The residue numbers of each region correspond to their positions in the S protein of MERS-CoV. HR2P, the peptide derived from the HR2 domain of MERS-CoV S protein S2 subunit; HR2P-M2, HR2P analogous peptide with mutations.

**Figure 4:**
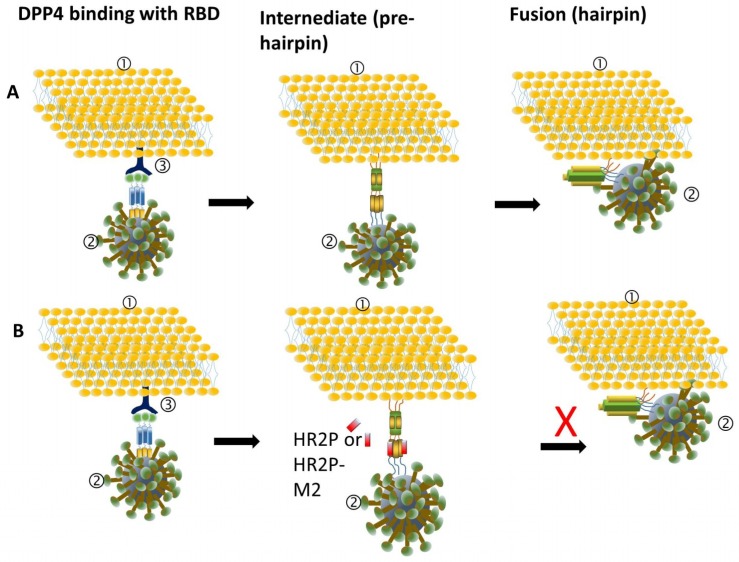
Schematic representation of the inhibition mechanism of HR2P and HR2P-M2. ① Target cell membrane; ② MERS-CoV; ③ dipeptidyl peptidase-4 (DPP4). (**A**) Mechanism of normal binding between a host cell and MERS-CoV. MERS-CoV enters the host cell by binding the viral particle via the RBD in spike protein to the cellular receptorDPP4 on the surface of the host cell. The HR2 binds to the HR1 to form a six-helix bundle (6-HB) fusion core, which brings viral and cell membranes into close apposition for fusion. (**B**) HR2P and HR2P-M2 block six-bundle fusion core formation between HR1 and HR2 peptides by binding to the viral S protein HR1 domain.

HR2P, spanning residues 1251–1286 in the HR2 domain, with low or no toxic effect in vitro, can effectively inhibit MERS-CoV replication by interacting with the HR1 domain to block spike protein-mediated cell–cell fusion and MERS-CoV pseudovirus entry (Table 1; Figure 3) [16]. To increase its stability, solubility, and anti-MERS-CoV activity, Lu et al. introduced a Glu, Lys, or Arg residue into HR2P, generating a new peptide, HR2P-M2 (Table 1). HR2P-M2 was indeed found to be more stable and soluble than HR2P. It blocked fusion core formation between HR1 and HR2 peptides by binding to the viral S protein HR1 domain and inhibiting S protein-mediated membrane fusion with an EC_50_ of 0.55 µM (Figure 4) [16,23]. HR2P-M2 is highly effective in inhibiting MERS-CoV infection in both Calu-3 and Vero cells with an EC_50_ of about 0.6 µM. Intranasal application of HR2P-M2 could significantly reduce the titers of MERS-CoV in the lung of Ad5-hDPP4 (adenovirus serotype-5–human dipeptidyl peptidase 4)-transduced mice [16,18]. Furthermore, intranasal administration of HR2P-M2 before viral challenge fully protected hDPP4-transgenic mice from MERS-CoV infection, whereas all untreated mice died 8 days after viral challenge [24]. Furthermore, by combining HR2P-M2 with interferon β, protection was enhanced for Ad5-hDPP4-transduced mice against infection by MERS-CoV strains with or without mutations in the HR1 region of the S protein, with >1000-fold reduction of viral titers in lung [18].

P21S10, the most effective fusion inhibitor of MERS-CoV, can inhibit MERS-CoV pseudovirus infection with an EC_50_ of about 1 µM in Huh-7 cells and a CC_50_ of >100 µM in Huh-7 cells by CCK8 (Cell Counting Kit-8) assay (Table 1) [20]. In addition, a series of synthesized stapled peptides, such as P21S10, P21S2, P21S4, P21S5, P21S8, P21S9, P21S8F, P21S8ZF, etc., could effectively inhibit infection by MERS-CoV pseudovirus and its spike protein-mediated cell fusion by blocking helix-mediated NHR (N-terminal heptad repeats) /CHR (C-terminal heptad repeats) interactions with a low EC_50_ and a high CC_50_ in Huh-7 cells [20].

P9, a short peptide, exhibited potent and broad spectrum antiviral effects against multiple respiratory viruses in vitro and in vivo [21,25]. P9 inhibited MERS-CoV with an EC_50_ of about 5 µg/mL in Madin-Darby canine kidney (MDCK) cells, obtained by plaque assay, and a CC_50_ of 380 µg/mL in MDCK cells obtained by MTT (3-(4,5-dimethyl-2-thiazolyl)-2,5-diphenyl-2-H-tetrazolium bromide) assay (Table 1) [21].

Lipopeptides are bioactive peptides that replicate the α-helical chain from the viral fusion machinery [22]. All 12 lipopeptides inhibit cell−cell fusion mediated by MERS-CoV S protein with EC_50_ values ranging from 0.1 to >10.0 µM in Huh-7 cells (Table 1) [22]. Among these lipopeptides, LLS and IIS were found to be the most potent MERS-CoV fusion inhibitors with EC_50_ values of 0.24 µM and 0.1 µM, respectively [22]. Other lipopeptides such as AAS, FFS, YYS, IIY, IIW, IIH, IIQ, IIK, and IIE can also inhibit cell−cell fusion mediated by MERS-CoV S protein with variable EC_50_ values [22].

**Figure 5:**
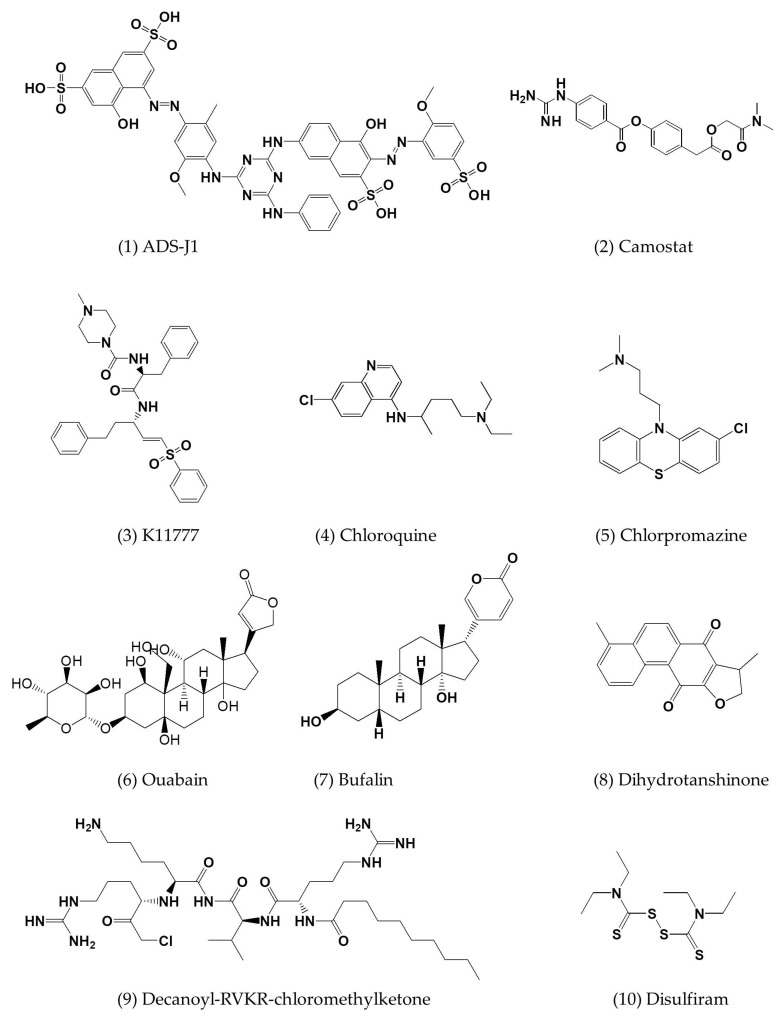

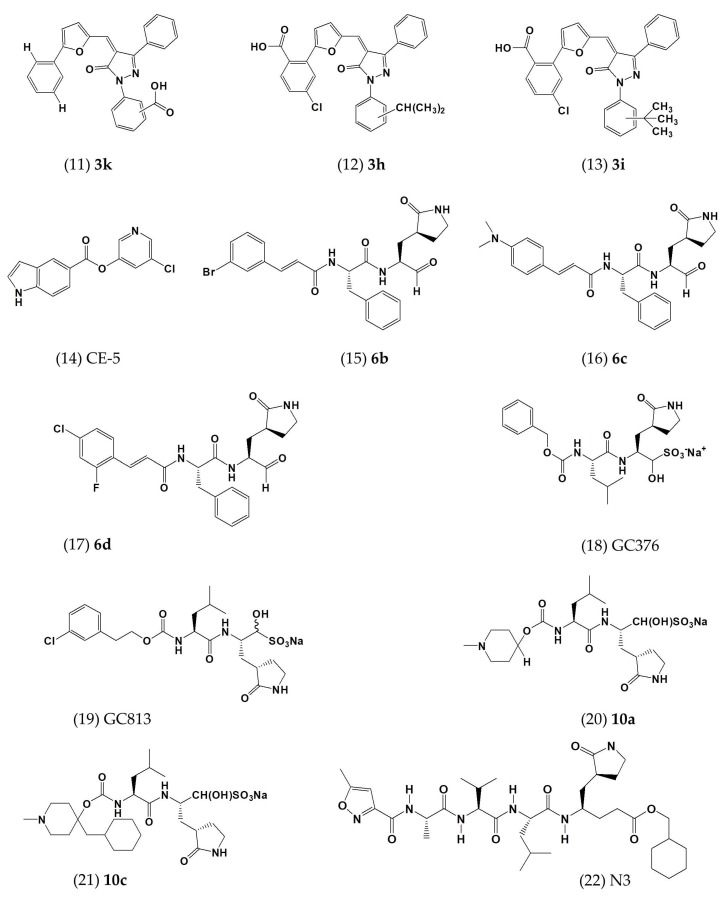

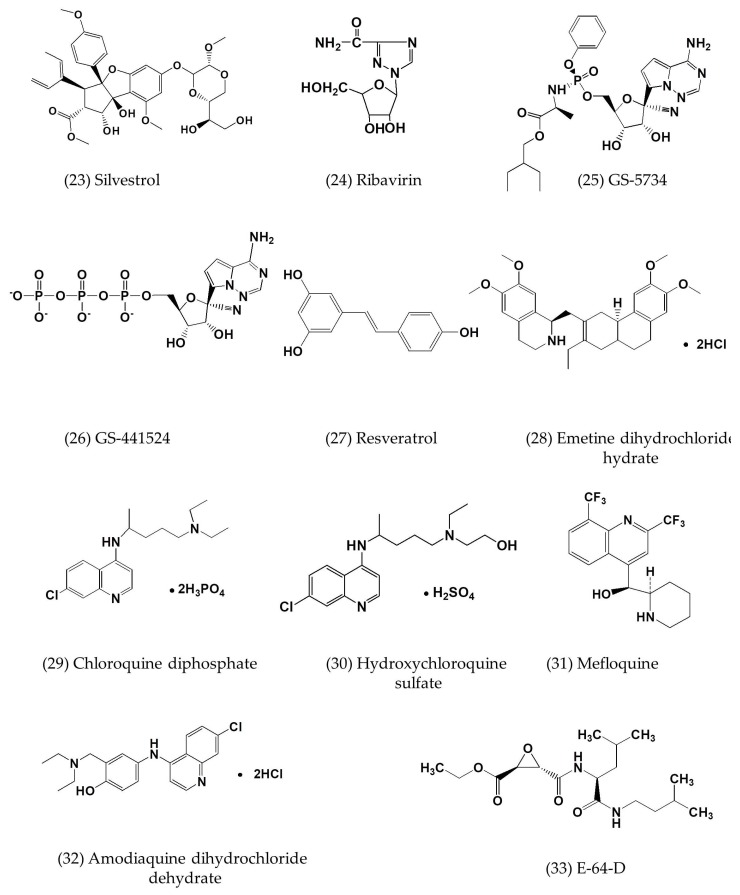

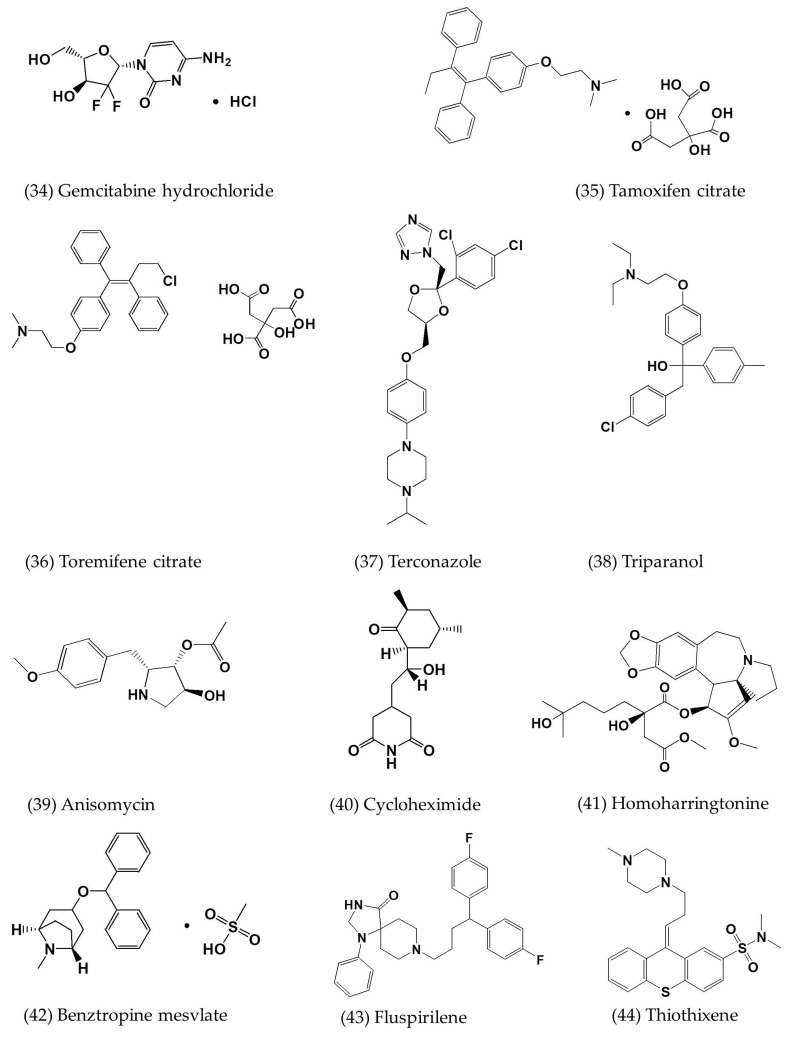

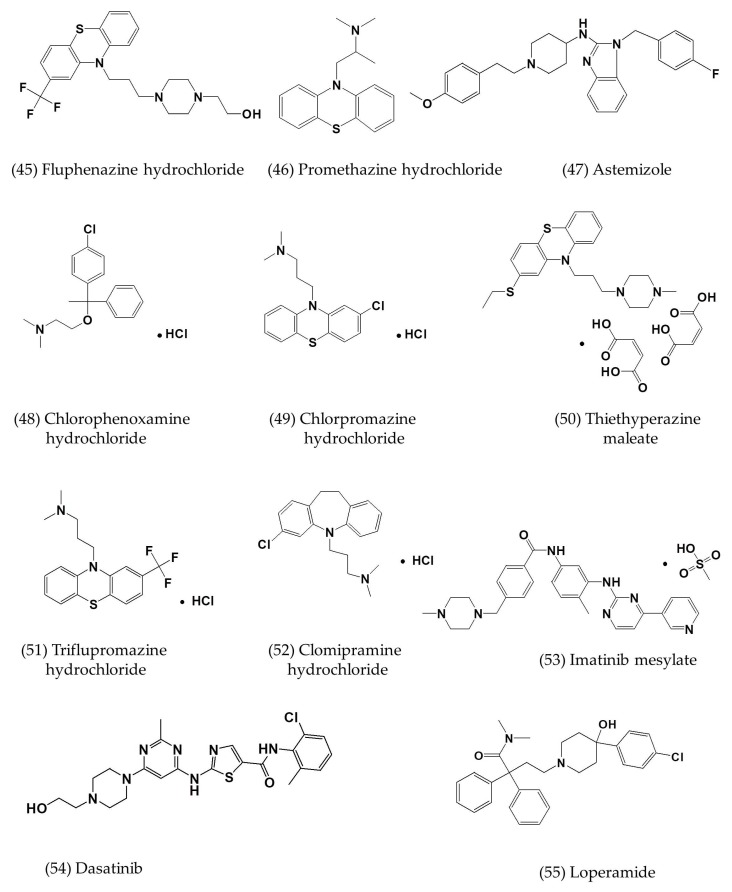

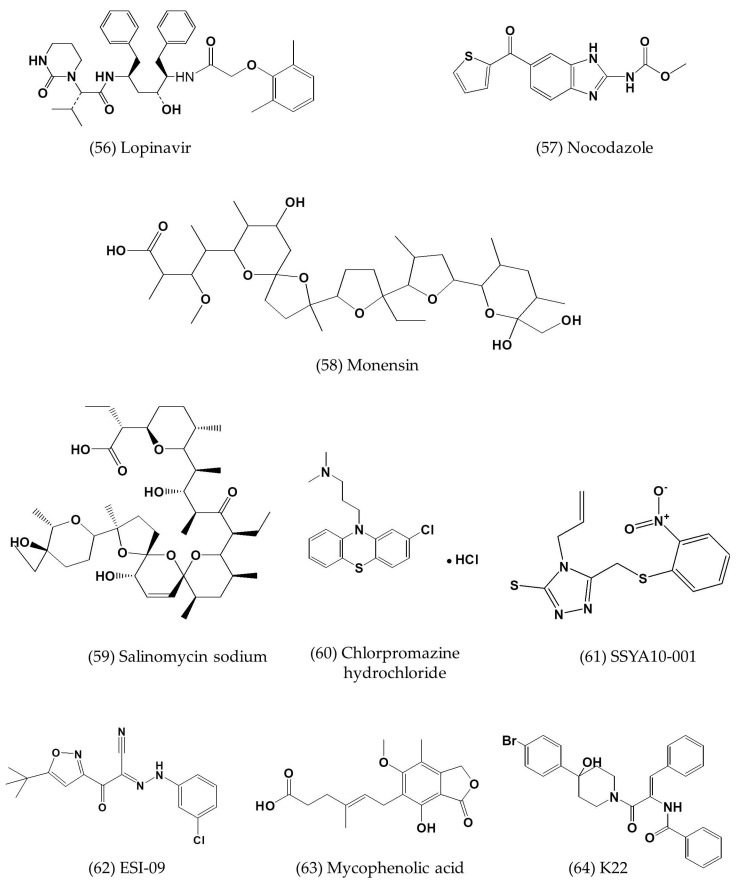

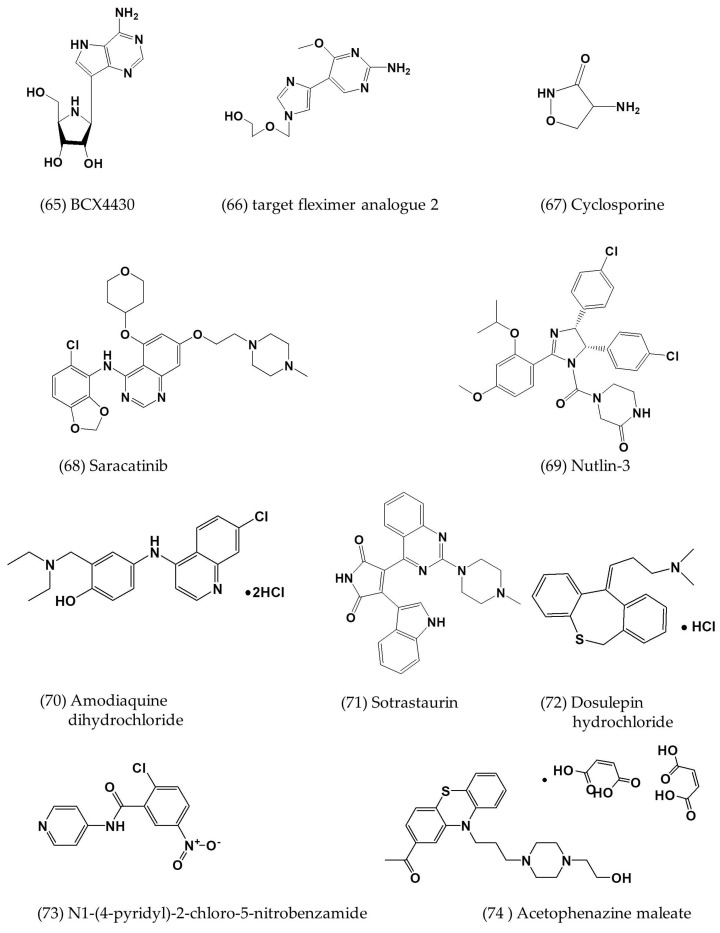

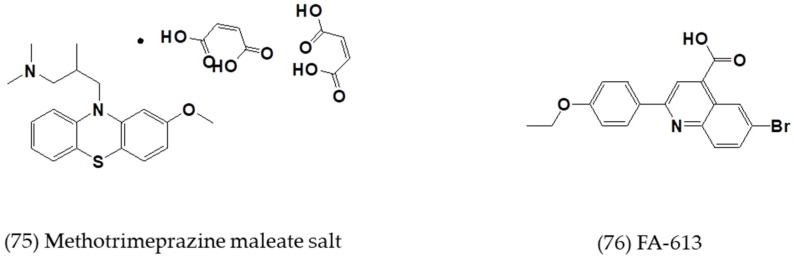
Chemical structure formulae of small-molecule inhibitors of MERS-CoV described in this review.

Three neurotransmitter inhibitors, including chlorpromazine, fluphenazine, and promethazine, were moderate inhibitors of cell–cell fusion with EC_50_ values of about 23, 15, and 17 µM, respectively (Table 2; Figure 5(5), (45), (46)) [26]. They can also disrupt clathrin-mediated endocytosis to inhibit MERS-CoV [26].

A small-molecule HIV entry inhibitor targeting gp41 ADS-J1 (Figure 5(1)) at the concentration of 20 µM could inhibit >90% of MERS-CoV pseudovirus infection in NBL-7 and Huh-7 cells. ADS-J1 could interrupt the interactions between the HR1 and HR2 of MERS-CoV to form the six-helix bundle, thus inhibiting the entry of pseudotyped MERS-CoV with an EC_50_ of 0.6 µM in the DPP4-expressing cell line and with a CC_50_ of 26.9 µM in NBL-7 and Huh-7 cells by MTT assay (Table 2) [27].

The elucidation of MERS-CoV interaction with its host cell is critical to the development of antiviral interventions. In order to gain entry into host cells, MERS-CoV not only uses DPP4 as a functional virus receptor, but also utilizes certain cellular proteases, such as TMPRSS2 and members of the cathepsin family, as activators of the S glycoprotein [9]. TMPRSS2 is expressed in epithelial cells of the human respiratory and gastrointestinal tracts [28,29,30,31]. The respective enzymes from host cells are also excellent targets for the identification of small-molecule MERS-CoV inhibitors. The serine protease inhibitor camostat mesylate (camostat) could completely block syncytium formation, but only partially block virus entry into TMPRSS2-expressing Vero cells (Figure 5(2)) [31].

K11777, a compound known to inhibit cruzain, a cathepsin-like protease from the protozoan parasite *Trypanosoma cruzi*, can inhibit MERS-CoV with an EC_50_ of 46 nM (Figure 5(3)) [32,33].

Chloroquine inhibited MERS-CoV replication and blocked infection at an early step with an EC_50_ of 3 µM and a CC_50_ of 58 µM (Table 2; Figure 5(4)) [34]. Chlorpromazine inhibited MERS-CoV replication at both early and post-entry stages with an EC_50_ of about 5 µM and a CC_50_ of 21 µM (Table 2; Figure 5(5)) [34]. However, high cytotoxicity narrowed the therapeutic window in both monocyte-derived macrophages (MDMs) and dendritic cells (MDDCs) [34].

Ouabain and bufalin can inhibit MERS-CoV entry by blocking clathrin-mediated endocytosis (Figure 5(6), (7)) [25,35]. The addition of small amounts of ouabain (50 nM) or bufalin (10 to 15 nM) inhibited infection with MERS-CoV and VSV (vesicular stomatitis virus) (Table 2), but only when the drug was added prior to inoculation in Huh-7 cells [35].

Dihydrotanshinone, a lipophilic compound, showed a decimal reduction at 0.5 µg/mL and excellent antiviral effects at ≥2 μg/mL with a reduction in titer from 6.5 Log to 1.8 Log TCID_50_/mL by using a pseudovirus expressing MERS-CoV spike protein (Figure 5(8)) [36].

During the biosynthesis of MERS-CoV S protein, the furin inhibitor decanoyl-RVKR-chloromethylketone (dec-RVKR-CMK) at 75 µM can lead to a decrease of the 85-kDa cleaved product in MERS-CoV S wt and S2′ mutant (Figure 5(9)) [37].

### 3.2. MERS-CoV Replication Inhibitors

#### 3.2.1. MERS-CoV Inhibitors Targeting Papain-Like Protease

Papain-like protease is a cysteine protease that uses the thiol group of cysteine as a nucleophile to attack the carbonyl group of the scissile peptide bond [38,39]. The genome of MERS-CoV encodes two polyproteins, ppla and pplb, which are processed by papain-like protease (PL_pro_) and 3C-like protease (3CL_pro_) [40]. MERS-CoV has only one papain-like protease, as does SARS-CoV, while other coronaviruses have two enzymes [41,42]. MERS-PL_pro_ is a part of the nonstructural protein nsp3, which includes three domains—namely, ubiquitin-like domain (UBL), a catalytic triad consisting of C1594–H1761–D1776, and the ubiquitin-binding domain (UBD) at the zinc finger—according to the homology model [40,43]. MERS-PL_pro_ is a multifunctional enzyme with deISGylating and deubiquitinating (DUB) activities [43], but it can also block the interferon regulatory factor 3 (IRF3) pathway [43,44].

Disulfiram, a drug used in alcohol aversion therapy, has been approved by the U.S. Food and Drug Administration (FDA) since 1951 (Figure 5(10)). It can inhibit the activity of some enzymes, such as urease [45], methyltransferase [46], and kinase [45], all by reacting with cysteine residues, suggesting broad-spectrum characteristics [47]. Notably, disulfiram also acts as an allosteric inhibitor of MERS-CoV papain-like protease [47]. Multiple inhibition assays also support a kinetic mechanism by which disulfiram, together with 6TG (6-thioguanine) and/or MPA (mycophenolic acid), can synergistically inhibit MERS-CoV papain-like protease [47]. Hence, the recombination of three clinically available drugs could feasibly be used to treat MERS-CoV infection.

#### 3.2.2. MERS-CoV Inhibitors Targeting 3C-Like Protease

The active site of MERS-3CL_pro_ can be divided into subsites S1–S6 [48]. Subsite S1 consists of vital catalytic residue Cys145 with His41 to process polyproteins at 11 conserved Gln sites, followed by small amino acids like Ala, Ser, or Gly [49]. Another crucial component of the S1 subsite is the oxyanion hole formed by the interaction of a carboxylate anion of conserved Gln with Gly143, Ser144, and Cys145, which stabilizes the transition state during proteolysis [50,51]. Glu166 at the entrance of the pocket interacts via H-bond with the Nɛ2 of the conserved Gln [50]. The S2 and S4 subsites contain hydrophobic and bulky side chains such as Val, Leu, or Phe. Subsites S5 and S6 are near the surface of the active site and have little participation in substrate binding [48].

Polyproteins pp1a and pp1b are processed by 3CL_pro_ (11 cleavage sites) and PL_pro_ (3 cleavage sites), resulting in 16 mature nonstructural proteins, including RNA-dependent RNA polymerase (RdRp) and helicase, which play important roles in the transcription and replication of coronaviruses [40,52]. Therefore, both proteases are essential for viral replication, making them attractive targets for drug development [52].

The analogues of hits of neuraminidase (NA) inhibitors on MERS-CoV 3CL_pro_ have been synthesized and showed average-to-good inhibition of MERS-3CL_pro_. The better one is the compound **3k** with an EC_50_ of 5.8 μM (Table 2; Figure 5(11)) [48]. Another two are compounds **3h** (Figure 5(12)) and **3i** (Figure 5(13)) with EC_50_ values of 7.3 and 7.4 µM, repsectively (Table 2) [48]. Furthermore, researchers have concluded that pharmacophores phenyl at R3 and carboxylate, either at R1 or R4, are essential for the antiviral activity [48]. Since the modification of rings A and B is well tolerated, these rings can be further altered to enhance the activity of the compounds. The SARS-CoV 3CL_pro_ inhibitor CE-5 can block the function of the MERS-CoV 3CL_pro_ (Figure 5(14)) [53]. Treatment with CE-5 inhibited the activity of MERS-CoV 3CL_pro_ to 30% of that of DMSO-treated cells at a maximum dose of 50 µM [53]. The endpoint evaluation of CE-5 indicated an EC_50_ of ~12.5 µM in cell culture (Table 2) [53].

Peptidomimetic inhibitors of enterovirus (**6b**, **6c**, and **6d**) inhibit MERS-CoV with EC_50_ values ranging from 1.7 to 4.7 µM, as shown by enzymatic assay (Figure 5(15), (16), (17)) [54]. As shown in Table 1, compounds **6b, 6c,** and **6d** efficiently suppressed viral replication with EC_50_ values of 1.4, 1.2, and 0.6 µM, respectively, after performing a cytopathic inhibition assay using MERS-CoV-infected Huh-7 cells (Table 2) [54].

GC376, a dipeptidyl transition state 3CL_pro_ inhibitor, can substantially inhibit the activity of MERS-CoV 3CL_pro_ with an EC_50_ of 1.6 µM by fluorescence resonance energy transfer (FRET) assay (Table 2; Figure 5(18)) [55].

GC813 as well as its synthesizing extended compounds **10a** and **10c** exhibit inhibition for MERS-CoV with EC_50_ values of 0.5 µM, 0.5 µM, and 0.8 µM in cell culture (Table 2; Figure 5(18), (19), (20), (21)) [52].

N3, a broad-spectrum anti-CoV inhibitor, can inhibit the proteolytic activity of MERS-CoV 3CL_pro_ by binding with the interface of domain I and II of MERS-CoV 3CL_pro_ with an EC_50_ of about 0.3 µM (Table 2; Figure 5(22)) [56].

### 3.3. Other Small-Molecule Inhibitors with Defined or Undefined Mechanisms of Action

Silvestrol, an eIF4A inhibitor, can inhibit MERS-CoV infection with an EC_50_ of 1.3 nM, as shown by plaque assay in MRC-5 cells and CC_50_ of 400 nM by MTT assay in peripheral blood mononuclear cells (PBMCs) (Table 2; Figure 5(23)) [57]. Silvestrol has broad-spectrum antiviral activity via the inhibition of the expression of CoV structural and nonstructural proteins (N, nsp8) and the formation of viral replication/transcription complexes [57].

The combination of interferon-α2b and ribavirin can effectively reduce MERS-CoV replication in vitro and in vivo (Table 2; Figure 5(24)) [6]. Rhesus macaques treated with IFN-α2b and ribavirin 8 h after MERS-CoV infection showed improved clinical parameters with no or very mild radiographic evidence of pneumonia compared with untreated macaques [6]. Moreover, treated macaques showed lower levels of systemic (serum) and local (lung) proinflammatory markers in addition to fewer viral genome copies, distinct gene expression, and less severe histopathological changes in the lungs [6].

GS-5734 (Remdesivir), the monophosphoramidate prodrug of the C-adenosine nucleoside analogue GS-441524, can inhibit the replication of the model β-coronavirus murine hepatitis virus (MHV) and RNA synthesis in wild-type (WT) virus, while an nsp14 ExoN (-) mutant lacking proofreading demonstrated increased susceptibility to GS-5734 (Figure 5(25)) [58]. GS-5734 also inhibits MERS-CoV infection with an EC_50_ of 0.074 ± 0.023 µM and a CC_50_ of >10 µM in human amniotic epithelial (HAE) cells (Table 2) [58]. Furthermore, GS-5734 acts at the early post-infection stage to decrease viral RNA levels, whereas delaying the addition of GS-5734 until 24 h post-infection resulted in decreased viral titer in HAE cell cultures at 48 and 72 h post-infection [58]. The nucleotide analogue GS-441524 also inhibits the infection of MERS-CoV with an EC_50_ of 0.9 µM and a CC_50_ of >100 µM in HAE cells (Table 2; Figure 5(26)) [58]. 

Resveratrol was found to significantly inhibit MERS-CoV infection as well as prolong cellular survival after virus infection (Figure 5. (27)) [66]. It was found that resveratrol could reduce RNA levels and infection titers in Vero cells [66]. Although resveratrol has minimal cytotoxicity, even at the high concentration of 250 μM, it can be ignored when compared to the much more severe toxicity of MERS-CoV infection [66].

A series of FDA-approved compounds were screened against MERS-CoV (Table 2) by cell-based ELISA assay (Figure 5(28–56)) [7]. Pharmaceuticals that inhibit MERS-CoV include neurotransmitter inhibitors, estrogen receptor antagonists, kinase signaling inhibitors, inhibitors of lipid or sterol metabolism, protein processing inhibitors, inhibitors of DNA synthesis/repair, as well as inhibitors of ion transport, cytoskeleton (specifically tubulin), and apoptosis [7]. Antiparasitics and antibacterials are two classes of pharmaceuticals, the functions of which are not obviously linked to coronaviruses, or viruses in general, but nonetheless show antiviral activity against MERS-CoV.

Nocodazole, targeting the cytoskeleton, specifically interferes with microtubule polymerization. It is an antimitotic drug developed for the treatment of cancer, but it was found to show high activity against MERS-CoV (Figure 5(57)) [67,68]. Monensin and salinomycin sodium, two of the nine ion channel inhibitors, have inhibitory activity against MERS-CoV, indicating that MERS-CoV may be susceptible to ionophore activities (Figure 5 (58), (59)). Chlorpromazine and chloroquine appear to target host factors, rather than viral proteins specifically, and the treatment of viral infections in patients aimed at host factors could reconfigure overt manifestations of viral pathogenesis into a less virulent subclinical infection and lower adverse disease outcome (Figure 5(60), (29)) [34,69].

Loperamide, an antidiarrheal opioid receptor agonist that reduces intestinal motility, also inhibits the replication of MERS-CoV at low-micromolar concentrations (3.3–6.3 µM) *in vitro* (Table 2; Figure 5(55)) [34]. Lopinavir, the HIV-1 protease inhibitor, inhibits MERS-CoV replication with an EC_50_ of 8 µM (Table 2; Figure 5(56)) [34].

SSYA10-001 inhibits MERS-CoV replication with an EC_50_ of ~25 μM in Vero E6 cells (Table 2; Figure 5(61)) [70]. Molecular modeling data suggest that SSYA10-001 can be docked with a comparable “Glide” score [70].

ESI-09 can reduce virus yield by inhibiting cAMP signaling in a cell type-independent manner (Figure 5(62)) [61]. The concentration of MERS-CoV inhibition by ESI-09 was found with an EC_50_ of 5 to 10 µM and a CC_50_ > 50 µM for both Calu-3 and Vero E6 cells by using the lactate dehydrogenase (LDH)-based cytotoxicity assay [62]. In addition, the undetectable cytopathic effect (CPE) and minimal expression of viral antigen indicated that Calu-3 cells treated with ESI-09 were almost fully protected [61].

Mycophenolic acid (MPA) can strongly reduce MERS-CoV replication by inhibiting inosine monophosphate dehydrogenase (IMPDH) and guanine monophosphate synthesis with an EC_50_ of 2.87 µM by cell-based ELISA in Vero E6 cells (Table 2; Figure 5(63)) [60].

K22 is a spectrum inhibitor which can inhibit MERS-CoV replication by reducing the formation of double membrane vesicles (DMVs) and by the near-complete inhibition of RNA synthesis (Figure 5(64)) [25,71].

BCX4430, an adenosine analogue that acts as a non-obligate RNA chain terminator to inhibit viral RNA polymerase function, can inhibit MERS-CoV infection with EC_50_ of 68.4 μM in Vero E6 cells by highly charged ions (HCIs)-based analysis and CC_50_ of >100 μM by neutral-red uptake (Table 2; Figure 5(65)) [25,62].

Fleximer nucleoside analogues of acyclovir are doubly flexible nucleoside analogues based on the acyclic sugar scaffold of acyclovir and the flex-base moiety in fleximers responsible for inhibiting RNA-dependent RNA polymerase (RdRp) [25,63]. The target fleximer analogue 2 can inhibit MERS-CoV infection with EC_50_ of 27 μM and CC_50_ of 149 μM in Huh-7 cells, but EC_50_ of 23 μM and CC_50_ of 71 μM in Vero cells (Table 2; Figure 5(66)) [63].

Interferon alpha1 (IFN-α1) and cyclosporine (CsA) have additive or synergistic effects in limiting MERS-CoV replication in ex vivo cultures of human bronchus (Figure 5(67)) [72]. In addition, the combined treatment of IFN-α1 and CsA has the most potent effect on inducing interferon-stimulated genes (ISGs) in both lung (24 hpi) and bronchial (56 hpi) tissues [72].

Saracatinib, a potent inhibitor of the Src-family of tyrosine kinases (SFK), potently inhibits MERS-CoV with an EC_50_ of about 3 μM in Huh-7 cells (Table 2; Figure 5(68)) [64]. It possibly inhibits MERS-CoV replication through the suppression of SFK signaling pathways at the early stages of the viral life cycle [64]. In addition, another seven compounds, primarily classified as antiprotozoal, anticancer, and antipsychotic, were also determined by complete dose-response analyses (Table 2; Figure 5(69–75)) [64].

A spectrum-inhibitor, FA-613, can inhibit MERS-CoV with an EC_50_ of ~10 μM in the interferon-competent cell line of Huh-7 cells, as shown by MTT assay (Table 2; Figure 5(76)) [65].

## 4. Strategies for Developing Small-Molecule MERS-CoV Inhibitors

The luciferase-based biosensor assay is a cell-based screening assay for selecting MERS-CoV-specific or broad-spectrum coronavirus PL_pro_ and 3CL_pro_ inhibitors [53]. HEK293T cells were transfected by two artificial plasmids: protease expression plasmids and biosensor expression plasmids [53]. Protease expression plasmids contain the sequence of MERS-CoV PL_pro_, the nonstructural proteins nsp4 and nsp5, as well as the N-terminal 6 region. Biosensor expression plasmids contain a circularly permuted *Photuris pennsylvanica* luciferase and the amino sequence of cleavage site of PL_pro_ or 3CL_pro_ [53]. After cell transfection and coexpression of a MERS-CoV protease domain with a cleavage-activated luciferase substrate, transfected live cells allow for both endpoint evaluation and live cell imaging profiles of protease activity [53]. This novel method can be performed in a biosafety level 2 research laboratory to evaluate the ability to inhibit the CoV protease activity of existing and new drugs [53].

Pseudovirus-based screening assays have been developed for identifying antiviral compounds in the MERS-CoV life cycle without using infectious viruses. The MERS-CoV pseudovirus allows for single-cycle infection of a variety of cells expressing DPP4, and results are consistent with those from a live MERS-CoV-based inhibition assay. More importantly, the pseudovirus assay can be carried out in a BSL-2, rather than a BSL-3 facility [9]. VSV- and HIV-luciferase pseudotyped with the MERS-CoV S protein are two more approaches [27].

Structure-Guided Design and Optimization of Small Molecules is a strategy that involves embodying a piperidine moiety as a design element to attain optimal pharmacological activity and protein kinase property [52]. This strategy permits the resultant hybrid inhibitor to participate in favorable binding interactions with the S3 and S4 subsites of 3CL_pro_ by attaching the piperidine moiety to a dipeptidyl component [52].

Ubiquitin-like domain 2 (Ubl2) is immediately adjacent to the N-terminus of the PL_pro_ domain in coronavirus polyproteins. In the past, the role of Ubl2 in PL_pro_ has remained undefined. However, evidence indicates that removing the Ubl2 domain from MERS PL_pro_ has no effect on its ability to process the viral polyprotein or act as an interferon antagonist, which involves deubiquitinating and deISGylating cellular proteins [73].

Analyzing the transcriptome of hosts infected with MERS-CoV can provide insight into how MERS-CoV infection influences and interacts with host cells. Josset et al. [74] infected a lung epithelial cell line, Calu3, with MERS-CoV and analyzed the transcriptome to identify inhibitory compounds resident in host factors that could be exploited as antiviral therapeutics. This approach can be used to identify host factors beneficial for virus propagation, thus establishing appropriate targets for existing or new antiviral inhibitors.

## 5. Conclusions

As a positive-sense, single-stranded RNA virus, MERS-CoV utilizes host cellular components to accomplish various physiological processes, including viral entry, genomic replication, and the assembly and budding of virions, thereby resulting in pathological damage to the host. Therefore, various stages of virus life cycle could be potential targets for developing small-molecule antiviral inhibitors. Inhibitors blocking MERS-CoV entry into host cells, viral protease inhibitors, and inhibitors targeting host cells and many other small-molecule inhibitors with defined or undefined mechanisms of action are summarized in this review.

Any compounds that interfere with virus infection may be harmful to host cells. Therefore, the establishment of a safety profile is essential. Furthermore, an antiviral inhibitor should effectively inhibit the growth of the virus because a small amount of virion replication can lead to resistant mutations. The advantages of small-molecule inhibitors include low price, stability, and the convenience of oral administration. Three main approaches are currently used to develop MERS-CoV small-molecule inhibitors. The first is the de novo synthesis of inhibitors targeting the unique structure in the proteins of MERS-CoV appearing in its infection process. The second approach involves screening inhibitors against MERS-CoV infection from an existing drug database by various chemical synthesis strategies. The third approach involves changing the chemical group of a fully developed drug to enhance its pharmacological activity against MERS-CoV. More novel strategies in improving the efficacy of screening small-molecule inhibitors are anticipated to reduce the threat of future MERS-CoV infections.

## Figures and Tables

**Table 1:** Peptide viral inhibitors against MERS-CoV.

Compound	Sequence	Testing Model	Cell Lines Tested	EC_50_ (μM)	CC_50_ (μM)	Ref.
**Peptide inhibitors disturbing membrane fusion**						
HR2P	SLTQINTTLLDLTYEMLSLQQVVKALNESYIDLKEL	In vitro	Vero cells Huh-7 cells	0.6 0.93 ± 0.15 ^b^	>1000	[16]
HR2P-M2	SLTQINTTLLDLEYEMKKLEEVVKKLEESYIDLKEL	In vitro; in vivo: hDPP4 Tg mice	Calu-3 and Vero cells; Ad5-hDPP4 mice	0.55 ± 0.04 ^b^	-	[16,18,19]
P21S10	LDLTYEM LSLQQVV K*LNE*Y	In vitro	Huh-7 cells	0.97 ± 0.08; 0.33 ± 0.04 ^b^	>100	[20]
P21S2	L*LTY*M LSLQQVV KALNESY	In vitro	Huh-7 cells	3.90 ± 1.1 ^b^	-	[20]
P21S4	LDLT*EM L*LQQVV KALNESY	In vitro	Huh-7 cells	7.14 ± 0.7 ^b^	-	[20]
P21S5	LDLTYEM *SLQ*VV KALNESY	In vitro	Huh-7 cells	10.7 ± 2.6 ^b^	-	[20]
P21S8	LDLTYEM LSLQ*VV K*LNESY	In vitro	Huh-7 cells	3.03 ± 0.29; 0.26 ± 0.05 ^b^	>100	[20]
P21S9	LDLTYEM LSLQQVV *ALN*SY	In vitro	Huh-7 cells	14.1 ± 2.3 ^b^	-	[20]
P21L2	LXLTYXM LSLQQVV KALNESY	In vitro	Huh-7 cells	10.9 ± 1.1 ^b^	-	[20]
P21L4	LDLTXEM LXLQQVV KALNESY	In vitro	Huh-7 cells	8.21 ± 0.9 ^b^	-	[20]
P21L5	LDLTYEM XSLQXVV KALNESY	In vitro	Huh-7 cells	4.49 ± 0.6 ^b^	-	[20]
P21L8	LDLTYEM LSLQXVV KXLNESY	In vitro	Huh-7 cells	20.6 ± 3.3 ^b^	-	[20]
P21L9	LDLTYEM LSLQQVV XALNXSY	In vitro	Huh-7 cells	10.9 ± 1.0 ^b^	-	[20]
P21L10	LDLTYEM LSLQQVV KXLNEXY	In vitro	Huh-7 cells	3.55 ± 0.2 ^b^	-	[20]
P21R8	LDLTYEM LSLQ^VV K^LNESY	In vitro	Huh-7 cells	16.3 ± 1.1 ^b^	-	[20]
P21S8Z	LDLTYEZ LSLQ*VV K*LNESY	In vitro	Huh-7 cells	2.80 ± 0.74; 0.63 ± 0.05 ^b^	>100	[20]
P21S8F	LDLTYEM LSLQ*VV K*LNESF	In vitro	Huh-7 cells	2.16 ± 1.1 ^b^	-	[20]
P21S8ZF	LDLTYES LSLQ*VV K*LNESF	In vitro	Huh-7 cells	3.89 ± 0.8 ^b^	-	[20]
P9 ^a^	NGAICWGPCPTAFRQIGNCGHFKVRCCKIR	In vitro	MDCK cells	5.00 μg/mL	380 μg/mL	[21]
LLS	LEELSKKLEELSKKLEELSKKLEELSKKLEELSKK-βA-K (C16)	In vitro	Huh-7 cells	0.24 ± 0.08 ^b^	4.04 ± 0.4	[22]
IIS	IEEISKKIEEISKKIEEISKKIEEISKKIEEISKK-βA-K (C16)	In vitro	Huh-7 cells	0.10 ± 0.02 ^b^	88.8 ± 28	[22]
AAS	AEEASKKAEEASKKAEEASKKAEEASKKAEEASKK-βA-K(C16)	In vitro	Huh-7 cells	4.47 ± 1.7 ^b^	2.38 ± 0.9	[22]
FFS	FEEFSKKFEEFSKKFEEFSKKFEEFSKKFEEFSKK-βA-K (C16)	In vitro	Huh-7 cells	3.11 ± 0.9 ^b^	>100	[22]
YYS	YEEYSKKYEEYSKKYEEYSKKYEEYSKKYEEYSKK-βA-K(C16)	In vitro	Huh-7 cells	6.26 ± 2.1 ^b^	19.8 ± 1.6	[22]
IIY	IEEIYKKIEEIYKKIEEIYKKIEEIYKKIEEIYKK-βA-K (C16)	In vitro	Huh-7 cells	0.52 ± 0.4 ^b^	>100	[22]
IIW	IEEIWKKIEEIWKKIEEIWKKIEEIWKKIEEIWKK-βA-K (C16)	In vitro	Huh-7 cells	10.6 ± 2.4 ^b^	>100	[22]
IIH	IEEIHKKIEEIHKKIEEIHKKIEEIHKKIEEIHKK-βA-K (C16)	In vitro	Huh-7 cells	1.68 ± 0.47 ^b^	>100	[22]
IIQ	IEEIQKKIEEIQKKIEEIQKKIEEIQKKIEEIQKK-βA-K (C16)	In vitro	Huh-7 cells	0.13 ± 0.1; 0.11 ± 0.02 ^b^	>100	[22]
IIK	IEEIKKKIEEIKKKIEEIKKKIEEIKKKIEEIKKK-βA-K (C16)	In vitro	Huh-7 cells	0.45 ± 0.13 ^b^	4.54 ± 0.6	[22]
IIE	IEEIEKKIEEIEKKIEEIEKKIEEIEKKIEEIEKK-βA-K (C16)	In vitro	Huh-7 cells	2.93 ± 0.95 ^b^	>100	[22]

^a^ P9-aci-1: three acidic amino acids D, E, and D were added to the C-terminus of P9. ^b^ Concentration of peptide that blocks MERS-CoV S-mediated cell–cell fusion. “-” indicates data not available. “*” indicates the position of the S5 residues, which react to form the all hydrocarbon staple. “^” indicates the positions of the R5 amino acids, which react to form staples. EC_50_: concentration for 50% of maximal effect. CC_50_: the 50% cytotoxicity concentrations.

**Table 2:** Small molecule viral inhibitors against MERS-CoV.

Inhibitor	Testing Model	Cell Lines	EC_50_ (μM)	CC_50_ (μM)	Ref.
**Inhibitors blocking the binding between virus and host cells**					
ADS-J1	In vitro	NBL-7 and Huh-7 cells	0.6	26.9	[27]
**Inhibitors disrupting endocytosis**					
Chlorpromazine	In vitro	Huh-7 cells	23.33 ± 2.89 ^a^; 49 ± 1.2; 9.514	>40; 21.3 ± 1.0	[5,6,7,26]
Promethazine	In vitro	Huh-7 cells	16.67 ± 7.22 ^a^; 11.802	>40	[7,26]
Fluphenazine	In vitro	Huh-7 cells	15.00 ± 4.33 ^a^; 5.868	~40	[7,26]
K11777	In vitro	Vero cells	0.046	>10	[32]
Camostat	In vitro	Vero-TMPRSS2 cells	~1	-	[31]
Ouabain	In vitro	Huh-7 cells	~0.05	-	[35]
Bufalin	In vitro	Huh-7 cells	0.01–0.015	-	[35]
Dihydrotanshinone	In vitro	-	0.5–1 μg/mL	-	[36]
**Inhibitors interrupting MERS-CoV MERS-CoV RNA replication and translation**					
Disulfiram	In vitro	-	22.7 ± 0.5	-	[47]
**3k**	In vitro	-	5.8 ± 1.6	-	[48]
**3h**	In vitro	-	7.3 ± 2.1	-	[48]
**3i**	In vitro	-	7.4 ± 2.2	-	[48]
CE-5	In vitro	HEK293T cells	~12.5	-	[53]
**6b**	In vitro	Huh-7 cells	1.4 ± 0.0	>100	[54]
**6c**	In vitro	Huh-7 cells	1.2 ± 0.6	>100	[54]
**6d**	In vitro	Huh-7 cells	0.6 ± 0.0	58.6 ± 1.2	[54]
GC376	In vitro	-	1.56 ± 0.09; 0.9	>150	[52,55]
GC813	In vitro	-	0.5	-	[52]
**10a**	In vitro	Vero81 cells	0.5	>100	[52]
**10c**	In vitro	Vero81 cells	0.8	>100	[52]
N3	In vitro	-	0.28 ± 0.02	-	[56]
**Inhibitors with undefined mechanisms**					
Silvestrol	In vitro	MRC-5 cells	0.0013	0.4	[57]
GS-5734	In vitro	HAE cells	0.074 ± 0.023	>10	[58]
GS-441524	In vitro	HAE cells	0.86 ± 0.78	>100	[58]
Chloroquine	In vitro	MDMs and MDDCs cells	3.0 ± 1.1; 6.275	58.1 ± 1.1	[7,59]
Emetine dihydrochloride hydrate	In vitro	Vero E6 cells	0.014	-	[7]
Hydroxychloroquine sulfate	In vitro	Vero E6 cells	8.279	-	[7]
Mefloquine	In vitro	Vero E6 cells	7.416	-	[7]
Amodiaquine dihydrochloride dehydrate	In vitro	Vero E6 cells	6.212	-	[7]
E-64-D	In vitro	Vero E6 cells	1.275	-	[7]
Gemcitabine hydrochloride	In vitro	Vero E6 cells	1.216	-	[7]
Tamoxifen citrate	In vitro	Vero E6 cells	10.117	-	[7]
Toremifene citrate	In vitro	Vero E6 cells	12.915	-	[7]
Terconazole	In vitro	Vero E6 cells	12.203	-	[7]
Triparanol	In vitro	Vero E6 cells	5.283	-	[7]
Anisomycin	In vitro	Vero E6 cells	0.003	-	[7]
Cycloheximide	In vitro	Vero E6 cells	0.189	-	[7]
Homoharringtonine	In vitro	Vero E6 cells	0.0718	-	[7]
Benztropine mesylate	In vitro	Vero E6 cells	16.627	-	[7]
Fluspirilene	In vitro	Vero E6 cells	7.477	-	[7]
Thiothixene	In vitro	Vero E6 cells	9.297	-	[7]
Astemizole	In vitro	Vero E6 cells	4.884	-	[7]
Chlorphenoxamine hydrochloride	In vitro	Vero E6 cells	12.646		[7]
Thiethylperazine maleate	In vitro	Vero E6 cells	7.865	-	[7]
Triflupromazine hydrochloride	In vitro	Vero E6 cells	5.758	-	[7]
Clomipramine hydrochloride	In vitro	Vero E6 cells	9.332	-	[7]
Imatinib mesylate	In vitro	Vero E6 cells	17.689	-	[7]
Dasatinib	In vitro	Vero E6 cells	5.468	-	[7]
Loperamide	In vitro	Vero E6 cells	4.8 ± 1.5	15.5 ± 1.0	[7]
Lopinavir	In vitro	Vero E6 cells	8.0 ± 1.5	24.4 ± 1.0	[7]
SSYA10-001	In vitro	Vero E6 cells	~25	>500	[60]
ESI-09	In vitro	Calu-3 and Vero E6 cells	5–10	>50	[61]
Mycophenolic acid	In vitro	Vero E6 cells	2.87	-	[60]
BCX4430	In vitro	-	68.4	>100	[62]
Fleximer analogues 2	In vitro	Vero cells Huh-7 cells	23 ± 0.6; 27 ± 0.0	71 ± 14; 149 ± 6.8	[63]
Nutlin-3	In vitro	Huh-7 cells	6.9 ± 1.4	26.8 ± 1.6	[64]
Amodiaquine dihydrochloride	In vitro	Huh-7 cells	2.1 ± 0.7	12.3 ± 5.9	[64]
Saracatinib	In vitro	Huh-7 cells	2.9 ± 0.6	57 ± 5.5	[64]
Sotrastaurin	In vitro	Huh-7 cells	9.7 ± 3.3	>50	[64]
Acetophenazine maleate	In vitro	Huh-7 cells	11.2 ± 5.0	23.6 ± 3.8	[64]
Dosulepin hydrochloride	In vitro	Huh-7 cells	3.4 ± 0.0	28.9 ± 0.0	[64]
Methotrimeprazine maleate salt	In vitro	Huh-7 cells	2.5 ± 0.0	24.5 ± 0.0	[64]
N1-(4-pyridyl)-2-chloro-5-nitrobenzamide	In vitro	Huh-7 cells	10.5 ± 0.3	>50	[64]
FA-613	In vitro	Huh-7 cells	10.2 ± 0.2	-	[65]

^a^ 50% effective concentration (EC_50_) values of inhibiting cell−cell fusion. “-” indicates data not available.
